# Modelling the epidemiology of malaria and spread of HRP2-negative *Plasmodium falciparum* following the replacement of HRP2-detecting rapid diagnostic tests

**DOI:** 10.1371/journal.pgph.0000106

**Published:** 2022-01-04

**Authors:** Alisha Chaudhry, Jane Cunningham, Qin Cheng, Michelle L. Gatton

**Affiliations:** 1 School of Public Health and Social Work, Queensland University of Technology, Brisbane, Australia; 2 Global Malaria Programme, World Health Organization, Geneva, Switzerland; 3 Department of Drug Resistance and Diagnostics, Australian Defence Force Malaria and Infectious Diseases Institute, Brisbane, Australia; 4 ADFMIDI Laboratory, QIMR Berghofer Medical Research Institute, Brisbane, Australia; 5 Centre for Immunology and Infection Control, Queensland University of Technology, Brisbane, Australia; Conservatoire National des Arts et Metiers, FRANCE

## Abstract

Malaria rapid diagnostic tests (RDTs) are dominated by products which use histidine-rich protein 2 (HRP2) to detect *Plasmodium falciparum*. The emergence of parasites lacking the *pfhrp2* gene can lead to high rates of false-negative results amongst these RDTs. One solution to restore the ability to correctly diagnose falciparum malaria is to switch to an RDT which is not solely reliant on HRP2. This study used an agent-based stochastic simulation model to investigate the impact on prevalence and transmission caused by switching the type of RDT used once false-negative rates reached pre-defined thresholds within the treatment-seeking symptomatic population. The results show that low transmission settings were the first to reach the false-negative switch threshold, and that lower thresholds were typically associated with better long-term outcomes. Changing the diagnostic RDT away from a HRP2-only RDT is predicted to restore the ability to correctly diagnose symptomatic malaria infections, but often did not lead to the extinction of HRP2-negative parasites from the population which continued to circulate in low density infections, or return to the parasite prevalence and transmission levels seen prior to the introduction of the HRP2-negative parasite. In contrast, failure to move away from HRP2-only RDTs leads to near fixation of these parasites in the population, and the inability to correctly diagnose symptomatic cases. Overall, these results suggest *pfhrp2-*deleted parasites are likely to become a significant component of *P*. *falciparum* parasite populations, and that long-term strategies are needed for diagnosis and surveillance which do not rely solely on HRP2.

## Introduction

Rapid diagnostic tests (RDTs) are a valuable and popular tool for malaria diagnosis with an estimated 2.7 billion RDTs being sold globally between 2010 and 2019 [1]. The large majority of RDTs for diagnosis of falciparum malaria contain antibodies to detect the *Plasmodium falciparum* histidine-rich protein 2 (HRP2). Indeed, 83 of the 85 malaria RDTs that have a specific *P*. *falciparum* test band and which met the WHO performance criteria (2013 to 2018) detect HRP2 [2].

HRP2-detecting RDTs are generally more sensitive than RDTs detecting other antigens since HRP2 is abundantly produced by *P*. *falciparum* parasites, is mostly released into patients’ blood when parasites rupture and ready for reinvasion, and accumulates in the plasma with a reported decay half-life between 3–5 days [3, 4]. However, it has been known for many years that the *pfhrp2* gene is not essential for parasite survival, with several long-term culture-adapted isolates showing deletions of *pfhrp2* [5, 6], and also deletions of the structurally similar *pfhrp3* gene [7].

Since the deployment of malaria RDTs for clinical diagnosis in the early 1990s there have been numerous reports of false negative RDT results [8–13]. There are a variety of potential reasons for false-negative RDT results, with the absence of the *pfhrp2* gene being one possibility [14]. Deletion of *pfhrp2* was first reported as an explanation for false negative RDT results in 2007 [15]. These *pfhrp2*-deleted isolates appeared to be constrained to the Amazon region of Peru, Columbia and Brazil where RDT use was limited and *P*. *vivax* is the dominant *Plasmodium* species. Retrospective analysis of stored samples showed that *pfhrp2*-deleted parasites have existed in this region since at least the late 1990s [16]. Prevalent false-negative RDTs among symptomatic patients with evidence of *pfhrp2-*deleted parasites have now been reported in a number of different countries [17]. An area of particular concern is the Horn of Africa, where recent reports demonstrate extremely high prevalence of *pfhrp2*-deleted parasites in Eritrea, Ethiopia and Djibouti [18–20].

Mathematical modelling has been used many times over to explore different aspects of malaria, ranging from the longevity and dynamics of infection within individuals, to the impact of interventions and drug resistance, to the cost-effectiveness of control or elimination activities. Agent-based models lend themselves to these types of activities, particularly in low transmission settings. A recent systematic review of agent-based models highlighted the diversity of published models, and a tendency for them to be tailored to answer specific questions [21]. A foundational building block in these models is the within-host dynamics, with different approaches and levels of complexity used to generate the infection dynamics [22]. Within the context of exploring the public health importance of HRP2*-*negative parasites, two specific models have been published showing the strong selection pressure created by the use of HRP2-only RDTs as the primary diagnostic tool for *P*. *falciparum* when *pfhrp2*-deleted parasites are introduced [23] or already present in the population [24]. Both these models were included in the systematic review of agent-based models [21], while the within-host model used by Gatton et al [23] is also examined by Camponovo et al [22].

The World Health Organization suggests deploying RDTs that do not exclusively rely on HRP2 where the prevalence of *pfhrp2*-deleted parasites causing false-negative RDT results in symptomatic patients with *P*. *falciparum* is at or above 5% [25]. Most of these alternate RDTs target *P*. *falciparum*-specific (Pf) or pan-species parasite lactate dehydrogenase (pLDH). While the rationale behind this approach is sound and should restore the ability to correctly diagnose *P*. *falciparum* infections and in theory remove the selection pressure for *pfhrp2*-deleted parasites, it is unknown what impact a switch in the type of RDT will have on the prevalence and spread of HRP2-negative parasites, and the overall transmission level within communities. Gatton et al [23] showed increased transmission and parasite carriage due to the spread of *pfhrp2-*deleted parasites using a simulation model. The work presented here investigates whether these trends are reversed following a switch to RDTs detecting pLDH, either alone or in combination with HRP2, and how the RDT change impacts on the prevalence of *pfhrp2-*deleted parasites and overall transmission level. The purpose is not to predict the time to key events, but rather compare the relative impacts of different decisions regarding when to switch to RDTs detecting pLDH.

## Methods

### Model

The agent-based stochastic simulation model previously reported by Gatton et al [23] was used to investigate the impact of switching the RDT used for diagnosis of symptomatic falciparum malaria in a village of 400 individuals. The model tracks the age, infection status, including details of each infecting parasite and circulating HRP2 and pLDH levels, and the immunological profile of each human in the village. Superinfection of humans by multiple parasites is accommodated in the model. Mosquito vectors are categorised as susceptible, infected or infectious, with the last two categories further defined according to the infecting parasite. Every parasite strain present in a host or mosquito is tracked and categorised according to its antigenic profile and HRP2 status. The village size used for the simulations has purposely been chosen to be small enough to satisfy the assumption of random mixing between hosts and vectors. Within this village setting, all antimalarial treatment comes from a single source and is provided only after confirmation of infection by RDT, and non-malarial fevers are not considered.

Previous use of this model indicated that although the values of some key outputs were sensitive to the mosquito parasites in the model, namely the proportion of mosquitoes feeding on humans and the probability of mosquitoes surviving a feeding cycle, the patterns in these key outputs over time were the same across the range of parameter values explored [23]. For this reason, only one set of mosquito parameter values were used in this study: the proportion of mosquitoes feeding on humans and the probability of mosquitoes surviving a feeding cycle were both assumed to be 0.5.

Within the model parasites were classified as either 1) HRP2-positive if they produced antigen detectable by a HRP2-detecting RDT, or 2) HRP2-negative if they produced no antigen detectable by a HRP2-detecting RDT. The assumed kinetics of HRP2 in HRP2-positive parasites are as previously reported [23], and it is assumed that both HRP2-positive and HRP2-negative parasites produce pLDH which decays as a first-order process with a half-life of 1.84 days [26]. The level of HRP2 and pLDH antigens within the host is monitored daily, as a combination of newly produced antigen and decaying antigen, and expressed as ‘equivalent parasites’ for each antigen. It is assumed HRP2 persists for a maximum of 21 days, compared to 15 days for Pf-LDH, meaning each antigen needs to be tracked independently. This approach results in the HRP2 antigen level being reflective of the HRP2-phenotype of all infecting parasites and their densities in the preceding 21 days.

RDTs used for diagnosis of symptomatic individuals in the model detected either HRP2 or pLDH, or both. The pLDH antigen could be either Pf-LDH or pan-LDH; hereafter only Pf-LDH RDTs are referred to but the results apply equally to pan-LDH RDTs if it is assumed that the antigen kinetics and RDT sensitivity are the same for Pf-LDH and pan-LDH.

It is assumed that RDTs detecting either antigen are always positive at >1,000 equivalent parasites/μL and always negative at <50 equivalent parasites/μL. Between these two values the probability of returning a positive result differs between antigens. For HRP2 the probability of a positive RDT is assumed to be 95% for equivalent parasite densities between 200 and 1000/μL, decreasing to 50% for equivalent parasite densities between 50 and 200/μL. For Pf-LDH RDTs the probability of a positive result is assumed as 75% and 10% for equivalent parasite densities between 200 and 1000, and 50 and 200 parasites/μL, respectively. This assumption mimics the general differences observed in performance between HRP2-based and LDH-based RDTs during the WHO Malaria RDT Evaluation Programme RDTs [2].

The simulated treatment following a positive diagnosis by RDT was artesunate-mefloquine, one of the WHO recommended artemisinin-based combination treatments (ACTs). It is assumed that parasites are sensitive to both artesunate and mefloquine, that the effect of each drug is additive, and that the artesunate component is active only for the first 3 days following treatment. The action of mefloquine was based on the model proposed by Simpon et al [27], with modification [23]. The proportion of parasites within a host surviving treatment was calculated as:

$$S_{i}=\left\{ \begin{array}{cc} {0.0001\times \{\frac{418.8^{2.5}+C_{i+2}^{2.5}}{418.8^{2.5}+C_{i}^{2.5}}\}}^{38.33} & for\;i<4\\ {\left\{\frac{418.8^{2.5}+C_{i+2}^{2.5}}{418.8^{2.5}+C_{i}^{2.5}}\right\}}^{38.33} & for\;4\leq i\leq 89\\ 1 & i>89 \end{array}\right.$$

$$C_{i}=1200e^{-0.036i},$$

where *S*_*i*_ is the proportion of asexual parasites surviving the replication cycle starting on the *i*^th^ day post-treatment.

Within the model parasite recombination was permitted when a feeding mosquito ingested gametocytes from different parasite isolates circulating within the infected host. This recombination involved creating new sets of *var* genes, a feature related to parasite antigenic variation and host immunity [23], as well as recombination between HRP2-positive and HRP2-negative parasites. This is a new feature in the model not previously reported. When a HRP2-positive male gametocyte mates with a HRP2-negative female gametocyte (or vice versa) the *pfhrp2* status of the newly recombined parasite is randomly selected with 50% chance of each genotype. It is assumed that there is no fitness gain or loss associated with *pfhrp2* gene status.

The model was programmed in Fortran and compiled using the Intel Fortran Compiler [28]. The program is executed via the Queensland University of Technology high-performance computing platform.

### Simulation scenarios

**Fig 1:**
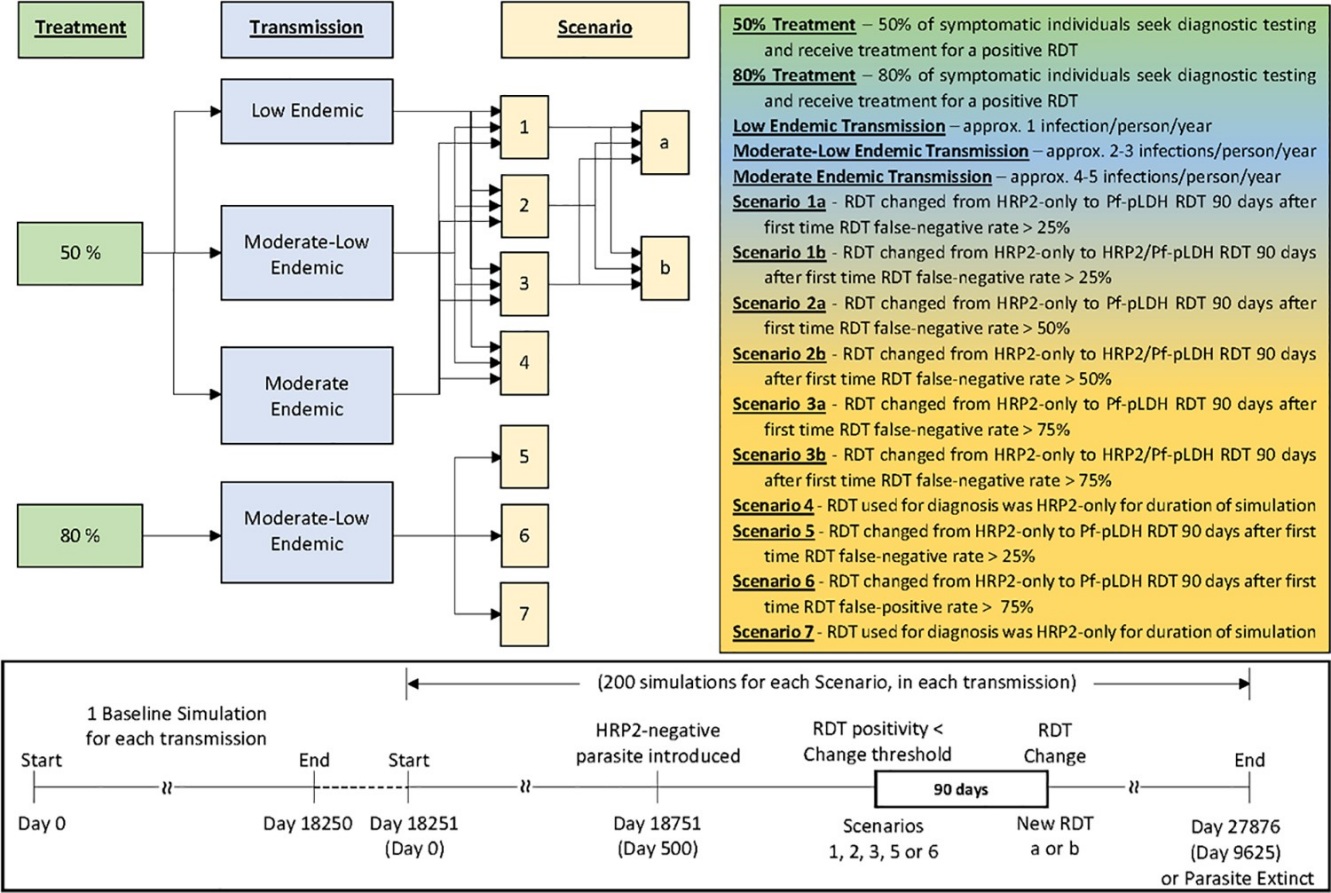
Flowchart outlining simulation methodology.

Three unique simulation sets were created. For each set a baseline simulation of 18,250 days (approximately 50 years) was conducted to establish stable transmission within the community and develop the immune profile of individuals. Genetically distinct, HRP2-positive parasites were introduced to the population every 50 days via a liver infection within a randomly selected host. Each simulation assumed 50% of symptomatic individuals sought diagnostic testing by HRP2-only RDT, and subsequent treatment if the RDT result was positive. The assumed treatment seeking rate of 50% is at the lower end of the rates reported globally [29]. The three simulation sets differed in the number of mosquitoes within the community, resulting in different transmission levels and epidemiological profiles at the end of the baseline simulation (Fig 1). Hereafter these simulation sets are referred to as low endemic (~1 infection/person/year), moderate-low endemic (~2–3 infections/person/year) and moderate endemic (~4–5 infections/person/year). Although the transmission level has been arbitrarily labelled, the entomological inoculation rate (EIR) is representative of that predicted for many endemic countries, particularly across Africa [30]. Previous modelling results of these transmission levels showed that at the end of the baseline simulation polyclonality was common with 19%, 54% and 80% of hosts infected with more than one isolate in the low, moderate-low and moderate settings, respectively [23]. The corresponding estimates for multiplicity of infection were 1.2, 1.5 and 2.7. The number of mosquitoes was relatively constant over time and no seasonality was included.

The baseline simulation formed the foundation for all other simulations within the set, such that subsequent simulations commenced using the baseline end conditions. The introduction rate of one new parasite every 50 days was maintained, with a single introduction of a HRP2-negative parasite 500 days into the simulations. All simulations initially used a HRP2-only RDT as the diagnostic. A switch in RDT to either a Pf-LDH RDT or a HRP2/Pf-LDH RDT was made in some simulations as described in the scenarios below, and in Fig 1. Two hundred simulations for each scenario were conducted.

The RDT false-negative rate was used to monitor RDT performance in the previous 30 days and is defined as the number of negative RDTs obtained from symptomatic patients infected with *P*. *falciparum* in the previous 30 days divided by the total number of RDTs tested on symptomatic patients infected with *P*. *falciparum* in the previous 30 days, multiplied by 100%. The only sources of false-negative RDT results in the model are RDT performance (based on parasite density) and HRP2-phenotype. The antigen levels associated with symptomatic infections typically exceeds 1,000 equivalent parasites/μL, the level where the assumed sensitivity of the RDTs is 100%. Therefore, the RDT false-negative rate in the model approximates the proportion of RDT results incorrectly classified as negative due to HRP2-neagtive *P*. *falciparum* among symptomatic individuals.

#### Scenario 1 (25% threshold)

The RDT was changed from a HRP2-only RDT to a Pf-LDH RDT (Scenario 1a) or a HRP2/Pf-LDH RDT (Scenario 1b) 90 days after the first occurrence of a RDT false-negative rate above 25% with at least 5 false-negative RDTs in the preceding 30 days.

#### Scenario 2 (50% threshold)

The RDT was changed from a HRP2-only RDT to a Pf-LDH RDT (Scenario 2a) or a HRP2/Pf-LDH RDT (Scenario 2b) 90 days after the first occurrence of a RDT false-negative rate above 50% with at least 5 false-negative RDTs in the previous 30 days.

#### Scenario 3 (75% threshold)

The RDT was changed from a HRP2-only RDT to a Pf-LDH RDT (Scenario 3a) or a HRP2/Pf-LDH RDT (Scenario 3b) 90 days after the first occurrence of a RDT false-negative rate above 75% with at least 5 false-negative RDTs in the previous 30 days.

#### Scenario 4

There was no change in the RDT used for diagnosis; a HRP2-only RDT was used for the duration of the simulation, irrespective of the RDT false-negative rate.

Another set of simulations was independently established using the same approach as described above but with the assumption that 80% of symptomatic individuals seek diagnostic testing and treatment, a treatment seeking rate at the higher end of those reported previously [29]. The transmission level at the end of the baseline simulation was classified as moderate-low endemic (~2–3 infections/person/year) and three scenarios were investigated, with 200 simulations in each (Fig 1):

#### Scenario 5 (25% threshold)

The RDT was changed from a HRP2-only RDT to a Pf-LDH RDT 90 days after the first occurrence of a RDT false-negative rate above 25% with at least 5 false-negative RDTs in the previous 30 days.

#### Scenario 6 (75% threshold)

The RDT was changed from a HRP2-only RDT to a Pf-LDH RDT 90 days after the first occurrence of a RDT false-negative rate above 75% with at least 5 false-negative RDTs in the previous 30 days.

#### Scenario 7

The RDT used for diagnosis was a HRP2-only RDT for the duration of the simulation, irrespective of the RDT positivity rate.

### Key outcomes from model

The key outcomes considered for each simulation were:

1. Establishment of the HRP2-negative parasite in the community, and subsequent survival after the RDT change. Parasites were classified as becoming established when they infected at least five individuals, in addition to the initial host. Parasites which became established to cause a change in RDT but did not survive until the end of the simulation period (25 years) were classified as becoming extinct.
2. RDT change day: the day the RDT was switched. This value was only calculated for scenarios involving a change of RDT (ie Scenarios 1, 2, 3, 5 and 6).
3. Proportion of asexual infections caused by HRP2-negative parasites: calculated every 50 days as the number of asexual infections (>10 parasites/μL) having a HRP2-negative phenotype divided by total number of asexual infections (>10 parasites/μL). A HRP2-negative phenotype was defined as having at least 10-fold higher density of HRP2-negative parasites compared to HRP2-positive parasites within the host.
4. Prevalence of infections with >100 parasites/μL and >1,000 parasites/μL: determined by a census of the population every 50 days and expressed as number of individuals with >100 parasites/μL and >1,000 parasites/ μL, respectively. The HRP2 phenotype of these infections was also recorded.
5. Proportion of asexual infections with a HPR2-negative phenotype that are high density: calculated every 50 days as the number of high density (>1,000 parasites/μL) asexual infections with a HRP2-negative phenotype divided by total number of asexual infections (>10 parasites/μL) with a HRP2-negative phenotype.
6. Number of treatments administered to symptomatic patients following a positive RDT result in the previous 50 days.
7. Entomological Inoculation Rate (EIR): calculated as the average number of infectious mosquitoes bites per person in the previous 50 days, scaled to represent an equivalent annual rate. The model assumes 60% of these infectious bites will initiate a new liver stage infection in the host [31].

### Statistical analysis

All statistical analysis was conducted using SPSS (IBM, Version 23). For each transmission level the proportion of simulations in which the initial HRP2-negative parasite become established were compared between scenarios using Pearson’s Chi-square test. General linear models were created to compare mean RDT change day for the 50% treatment scenarios (Scenarios 1 to 3 at three transmission levels), while a t-test was used to compare the mean RDT change day between Scenarios 5 and 6.

Binary logistic regression was applied for each transmission level to determine whether the type of RDT used after the RDT switch influenced the odds of extinction, adjusting for RDT change threshold. Kaplan-Meier survival curves were used to visualise the time to extinction, while Cox proportional hazards regression was used to determine whether there were differences in the hazard of extinction following an RDT change, with type of RDT used after the switch and RDT change threshold as factors.

Due to the stochastic nature of the simulations, values for key measures such as prevalence, number of treatments and EIR fluctuate over time. Within the model each of these parameters is reported every 50 days. To allow comparison of trends in the parameters a reference value for each parameter was created using the 75^th^ percentiles reported during the 500 days prior to the introduction of the HRP2-negative parasite, for each simulation. The 75^th^ percentile was selected as the threshold representing the upper level observed for the majority of simulations. This provided a clear benchmark for comparison before and after the introduction of the HRP2-negative parasite, without over-inflating the baseline values. Hence multiple simulation trajectories above this benchmark post-introduction signaled increased prevalence or treatment or EIR.

## Results

### Establishment of the HRP2-negative parasite in population

Overall 82.5% (1,155/1,400), 76.9% (1,077/1,400) and 62.2% (871/1,400) of HRP2-negative parasite introductions became established in the low, moderate-low and moderate endemic simulations when 50% of symptomatic patients sought diagnostic testing and treatment. Within each transmission level there was no significant difference in establishment rates between different RDT change scenarios (S1 Table). A higher proportion (89.7%; 538/600) of introductions became established in the moderate-low endemic setting when 80% of symptomatic patients sought diagnostic testing and treatment, with no significant difference between the three different scenarios (p = 0.141).

### Timing of RDT switch in Scenarios 1, 2, 3, 5 and 6

All simulations where the HRP2*-*negative parasite became established subsequently met the threshold conditions for a RDT switch. Median RDT change day ranged from 332 and 327 for Scenario 1 in low and low-moderate transmission, respectively, to 1,610 days for Scenario 3 in the moderate transmission setting (S2 Table). For Scenarios 1, 2 and 3, within each transmission level the day the RDT was changed was associated with the change threshold (p<0.001), and the RDT change day increased with increasing transmission level (p<0.001, S2 Table). A similar pattern was observed for Scenarios 5 and 6 (80% treatment seeking scenarios) with the mean time between introduction of the initial HRP2-negative parasite and the RDT switch increasing from 241.6 days (95% CI: 235.3–248 days) for Scenario 5 to 333.3 days (95% CI: 326.3–340.2 days) for Scenario 6 (p<0.001).

### Dynamics of HRP2-negative parasites with continued use of HRP2-only RDT (Scenario 4)

Following the introduction of the HRP2-negative parasite, high density infections were found to have a relatively high risk of being caused by HRP2-negative parasites; in the 250 days following introduction, high density infections (>1,000/μL) were on average 4.1 to 5.3 times more likely to contain the HRP2-negative phenotype than low density (10 - ≤1,000/μL) infections. This relative risk decreased to an equilibrium value of 1.0 after approximately 800 days signaling the end of the predominance for the HRP2-negative phenotype to cause high density infections.

**Fig 2:**
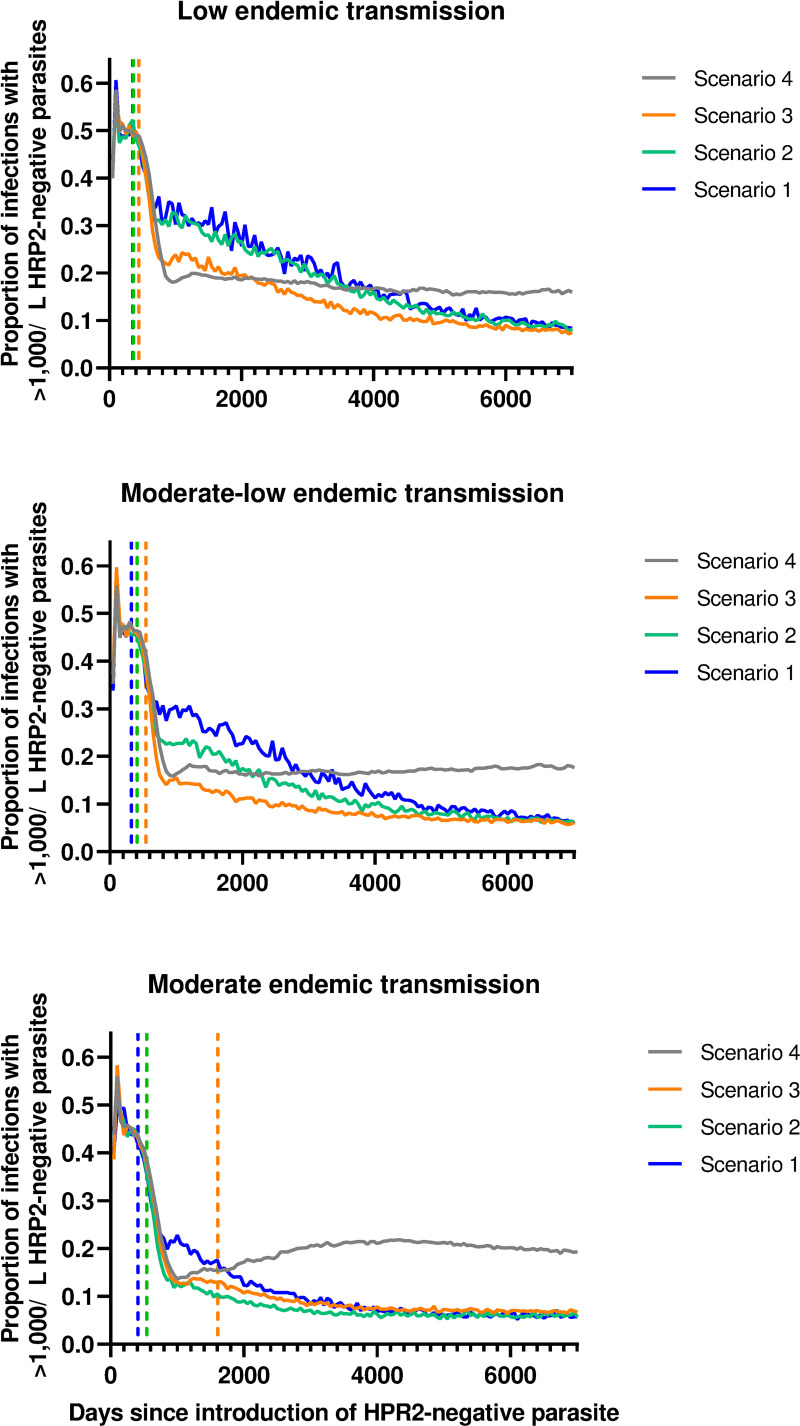
Average proportion of all infections (>10/μL) containing the HRP2-negative phenotype which were high density (>1,000/μL). Proportions are calculated within individual simulations, then averaged across simulations. Vertical reference lines indicate the median RDT change day for Scenarios 1, 2 and 3.

During the first 500–600 days following introduction, more than 40% of infections containing HRP2-negative parasites were high density (>1,000/μL) (Fig 2). This proportion rapidly declined to approximately 20% by 1,000 days post-introduction, irrespective of transmission intensity.

**Fig 3:**
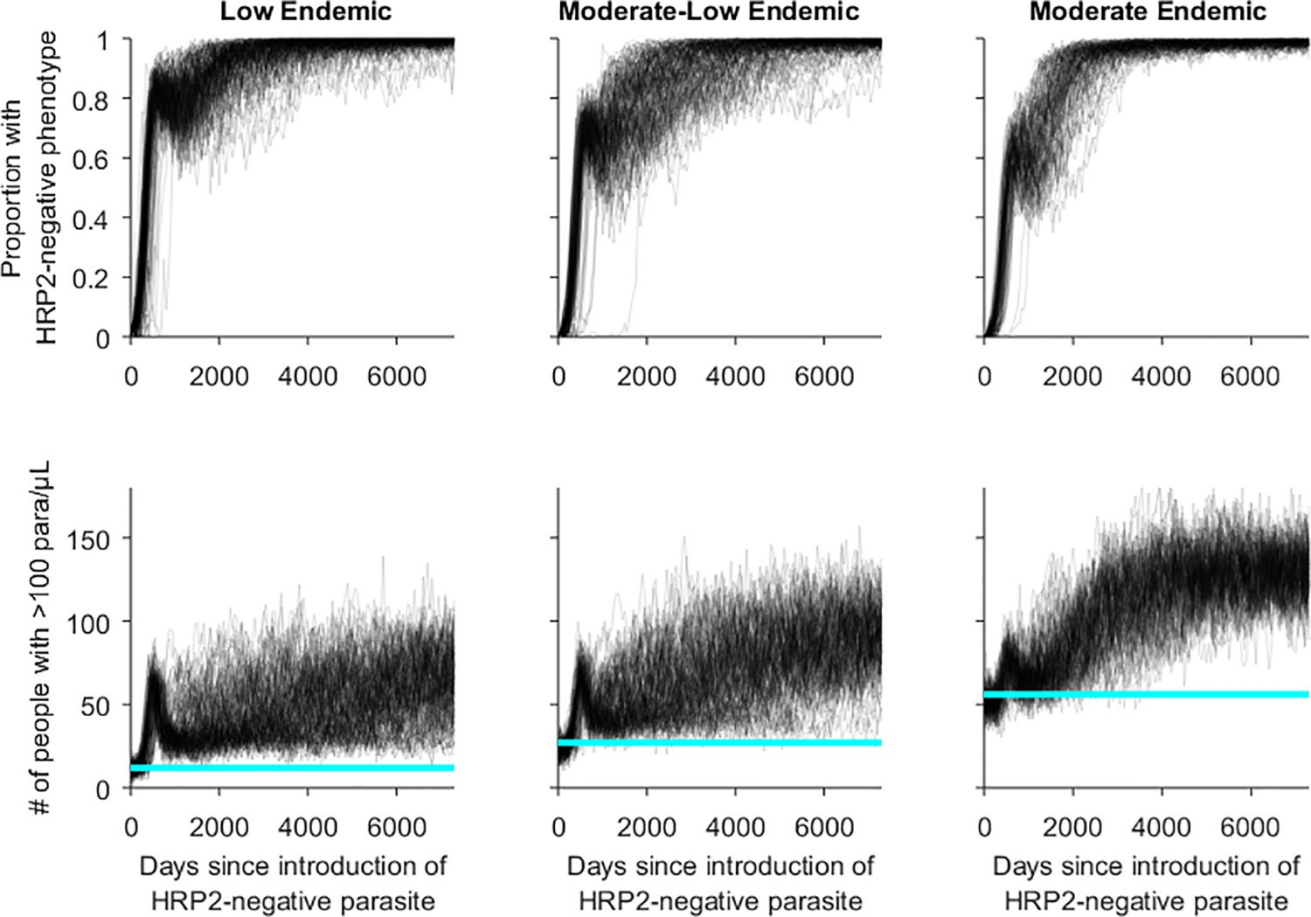
Simulation trajectories for the proportion of all asexual infections (>10 parasites/μL) with a HRP2-negative phenotype (top), and prevalence of asexual infections (>100 parasites/μL) (bottom) where 50% of symptomatic patients seek diagnosis and treatment using a HRP2-only RDT (Scenario 4). Horizontal reference line: 75^th^ percentile of prevalence in 500 days before introduction of HRP2-negative parasite.

Over the simulation period the actual proportion of all infections containing a HRP2-negative phenotype increased to near saturation of the parasite population within 20 years, with a corresponding increase in the number of people infected (Fig 3).

### Dynamics of HRP2-negative parasites following RDT switch

Overall, between 1.1% and 54.5% of simulations demonstrated extinction of the HRP2-negative parasite following the RDT switch (Table 1). The odds of extension of HRP2-negative parasites decreased as the RDT change threshold increased (Table 1). The analysis was not conducted for Scenarios 5 or 6 as too few simulations (19/185, 10.3% for Scenario 5 and 2/180, 1.1% for Scenario 6) resulted in the HRP2*-*negative parasite becoming extinct.

**Table 1:** Extinction of HRP2-negative parasites following RDT change.

Transmission	Scenario	Proportion of simulations where HRP2-negative parasite became extinct (n)	Odds Ratio (p-value)
Low endemic	1 (25% threshold)	0.502 (323)	Ref
	2 (50% threshold)	0.321 (333)	0.471 (p<0.001)
	3 (75% threshold)	0.114 (334)	0.128 (p<0.001)
Moderate-low endemic	1 (25% threshold)	0.545 (308)	Ref
	2 (50% threshold)	0.175 (315)	0.176 (p<0.001)
	3 (75% threshold)	0.067 (298)	0.06 (p<0.001)
Moderate endemic	1 (25% threshold)	0.353 (249)	Ref
	2 (50% threshold)	0.136 (235)	0.289 (p<0.001)
	3 (75% threshold)	0.012 (252)	0.022 (p<0.001)

The type of RDT used after the RDT switch did not affect the hazard of extinction (p>0.093), but the triggering threshold for the RDT change did (p<0.001). Extinction was more common, and occurred earlier, for Scenario 1 (25% threshold), followed by Scenario 2 (50% threshold) then Scenario 3 (75% threshold) (S1 Fig).

**Fig 4:**
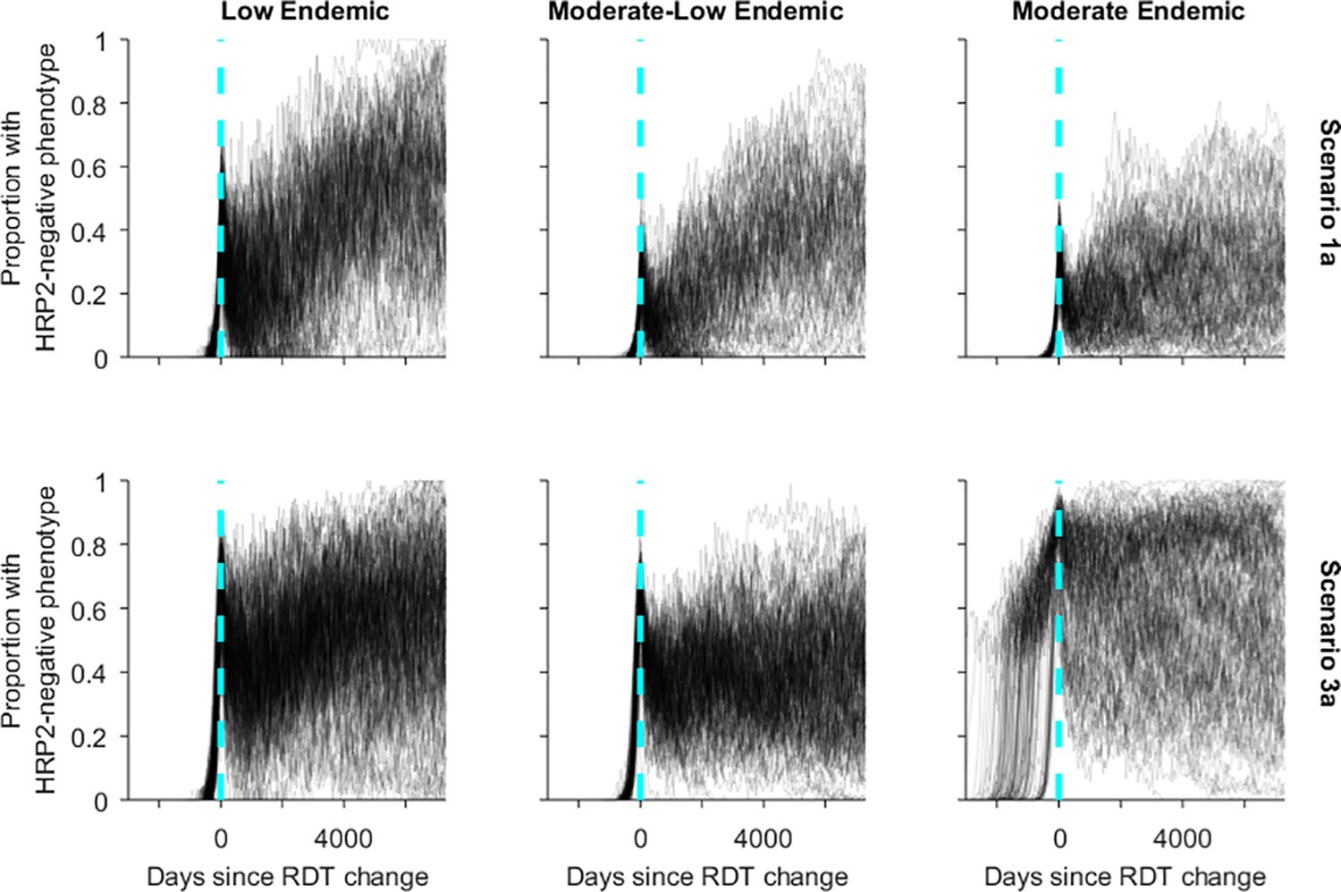
Simulation trajectories for the proportion of all asexual infections (>10 parasites/μL) with a HRP2-negative phenotype where 50% of symptomatic patients seek diagnosis and treatment and a Pf-LDH RDT is used after the RDT change. Vertical line represents RDT change day.

Following the RDT switch there was large variability in the proportion of infections caused by HRP2-negative parasites within individual simulations. Many simulations showed an increase in the proportion of infections with a HRP2-negative phenotype following the RDT switch, while others experienced a decline (Fig 4, S2–S4 Figs).

An earlier RDT switch (Scenario 1) resulted in the proportion of HRP2-negative parasites in high density infections remaining elevated for longer compared to when no RDT switch was made (Scenario 4) (Fig 2), irrespective of transmission intensity. However, in the long-term any RDT switch resulted in a lower proportion of HRP2-negative parasites in high density infections compared to no RDT switch (Scenario 4).

### Impact of RDT switch on prevalence and transmission intensity

#### 50% treatment seeking rate (Scenarios 1 to 3)

**Fig 5:**
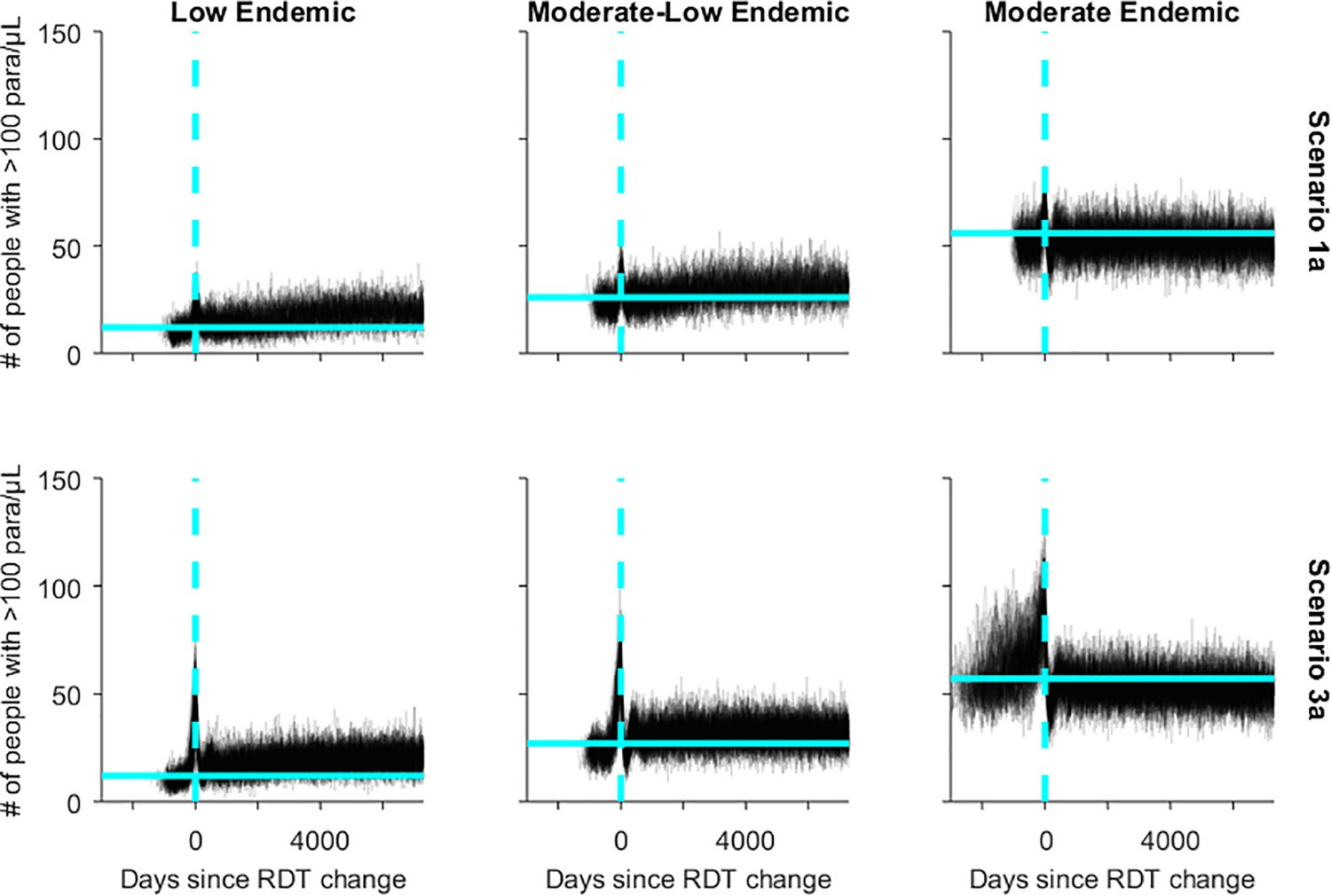
Simulation trajectories for prevalence of asexual infections (>100 parasites/μL) in a village where 50% of symptomatic patients seek diagnosis and treatment and a Pf-LDH RDT is used after the RDT change. Vertical dashed line: RDT change day; light blue horizontal: 75^th^ percentile of prevalence in 500 days before introduction of HRP2-negative parasite.

The long-term impact of the HRP2-negative parasite on prevalence (>100 parasites/μL) of infection and transmission intensity was assessed by comparing trends over time against the 75^th^ percentiles in the 500 days prior to the introduction of the HRP2-negative parasite. Just prior to the RDT switch all simulations, particularly those in Scenarios 2 and 3, showed a steep increase in the number of infected individuals. The switch in RDT was immediately followed by a drop in the prevalence of infection, however the long-term prevalence tended to remain above (low endemic and low-moderate endemic) or fluctuate around (moderate endemic) the pre-introduction values (Fig 5 and S5 Fig). In all cases the prevalence following the RDT switch was lower than in the simulations in which the RDT was not changed (Fig 3).

**Fig 6:**
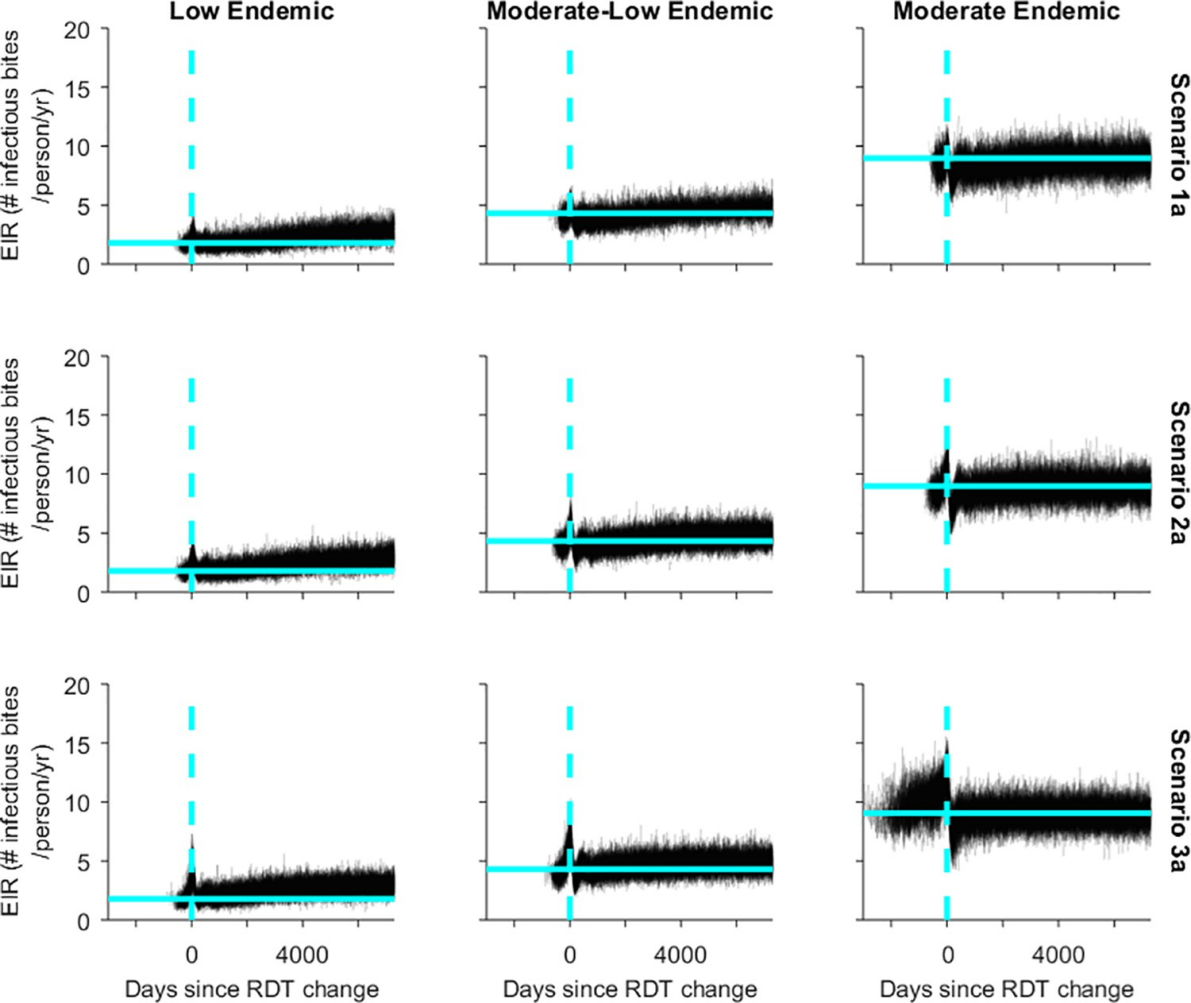
Simulation trajectories for EIR in a village where 50% of symptomatic patients seek diagnosis and treatment and a Pf-pLDH RDT is used after the RDT change. Vertical dashed line: RDT change day; light blue horizontal line: 75^th^ percentile of EIR in the 500 days before introduction of HRP2-negative parasite.

In the low and moderate-low endemic settings the EIR gradually increased during the simulation period (Fig 6). This pattern was not observed in simulations of the moderate endemic setting, with the EIR fluctuating around the 75^th^ percentile recorded in the 500 days prior to the introduction of the HRP2-negative parasite (Fig 6).

#### 80% treatment rate (Scenarios 5 to 7)

**Fig 7:**
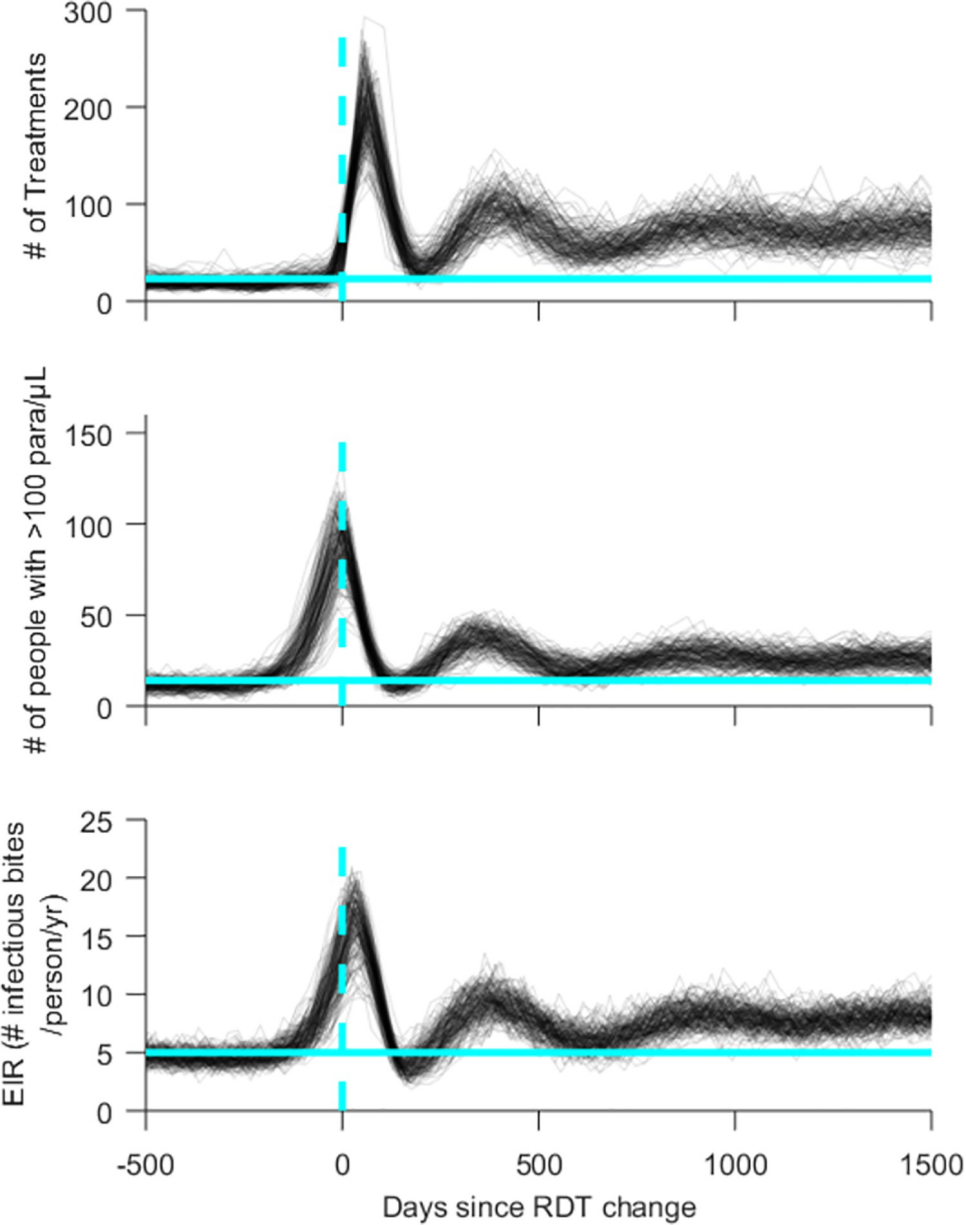
Simulation trajectories for number of treatments in previous 50 days, prevalence (>100 parasites/μL) and EIR in a village with moderate-low transmission where 80% of symptomatic patients seek diagnosis and treatment and a Pf-pLDH RDT is used after the false-negative RDT rate exceeds 75% (Scenario 6). Vertical dashed line: RDT change day; horizontal line: 75^th^ percentiles of parameters in 500 days before introduction of HRP2-negative parasite.

Simulations of the moderate-low endemic transmission level where 80% of symptomatic individuals seek diagnosis and treatment showed continued elevated prevalence and transmission following the RDT switch (S6 Fig). The simulation trajectories for the first 1000 days following the RDT change also showed clear oscillations with decreasing amplitude for the number of treatments, prevalence and EIR (Fig 7).

## Discussion

The emergence of HRP2-negative parasites represents a significant impediment to the diagnosis of falciparum malaria by RDT due to the dominance of HRP2 as the target antigen. Previous modelling studies have shown that the use of HRP2-detecting RDTs for diagnosis of falciparum malaria in areas where *pfhrp2*-deleted parasites exist can provide strong selection pressure, leading to high false-negative rates in symptomatic patients [23, 24]. The results from this study reinforce those findings and clearly show that failure to move away from using HRP2-only RDTs once *pfhrp2*-deleted parasites are established leads to increased prevalence and transmission.

However, while changing away from a HRP2-only RDT generally prevented HRP2-negative parasites from reaching fixation, it did not always result in extinction of the HRP2-negative parasites. Prevalence and transmission were generally reduced after the RDT switch, although their levels often remained higher than before the HRP2-negative parasite introduction. These results may be explained by the long-term trend for only approximately 10% of HRP2-negative infections to be high density (>1,000/uL), with only a proportion of these likely to be symptomatic. Thus, the majority of HRP2-negative parasites are predicted to be circulating at lower densities in asymptomatic individuals. This reflects the patterns reported from the DRC where children infected with *pfhrp2*-deleted parasites were more likely to be microscopy-negative and afebrile [32].

Importantly, the model results suggest that delays in changing away from a HRP2-only RDT once the HRP2-negative parasite is established reduce the chances of the HRP2-negative phenotype becoming extinct, or being able to reduce transmission back to baseline values. The WHO recommends moving away from HRP2-only RDTs in areas where *pfhrp2*-deleted parasites have been confirmed to exist and are causing >5% false-negative rate in symptomatic patients [25]. However, in field settings there is no constant surveillance to indicate when this threshold is crossed in real time, and there are operational difficulties associated with effecting a change in diagnostic test (eg order and acquisition time, re-training of health workers) that delay the implementation of the new diagnostic. Our modelling study uses much higher threshold levels due to the small population size and the desire to demonstrate trends in change thresholds above and beyond the stochastic variation within the model. Irrespective of the RDT change threshold, it was clear that false-negative RDTs tend to emerge earlier after the introduction of HRP2-negative parasites in lower transmission settings, suggesting these may be the regions to prioritize for surveillance and first trigger action for a RDT change. The continued presence of HRP2-negative parasites within a community for many years following the RDT change potentially has long-term negative impacts on the use of this antigen for screening for severe malaria [33, 34] and active case detection in elimination efforts [35].

There was large variation in the prevalence of HRP2-negative parasites following the RDT change due to the stochastic components within the model. In some simulations HRP2-negative parasites became extinct, while in others they continued to spread and dominate the parasite population. One possible explanation may relate to the relative homogeneity of *pfhrp2*-deleted parasites [18], and the acquisition of clinical immunity. The rapid spread of the initial HRP2-negative parasite and failure to treat subsequent infections creates an opportunity for many hosts to develop clinical immunity against this specific parasite. This results in lower density infections, and potentially multiple infections and subsequent parasite recombination between HPR2-negative and HRP2-positive parasites. The new parasite will share 50% of the *var* gene repertoire, a repertoire against which many hosts within the community potentially have some immunity. It is plausible that inheriting 50% of the *var* genes creates partial immunity, thereby decreasing the chances of clinical symptoms and treatment, further increasing the opportunity for multi-clone infections within a host. The decrease in the proportion of HRP2-negative parasites in high density infections supports this hypothesis.

Another interesting result is the oscillations in treatment, prevalence and EIR following the RDT change at the higher treatment rate. These oscillations were also observable at the lower treatment rate but were not as pronounced. The oscillating nature of the association can be attributed to the high treatment rate following the RDT change causing a significant number of potential hosts within the community to be protected from reinfection by the long-acting mefloquine component of the ACT. This in turn reduces transmission, impacting the EIR. As the prophylactic effect of mefloquine declines over time multiple hosts become susceptible to reinfection at approximately the same time, creating a new but smaller wave of treatment as not every infected host will become symptomatic or seek treatment. Such patterns also occur following mass drug administration or other short-lived treatments, with the effect size related to the number of hosts treated.

An unexpected result from this study was the trend for continued increased prevalence and transmission following the RDT change, particularly in the low endemic setting. This pattern occurred irrespective of whether the new RDT detected Pf-LDH only, or HRP2/Pf-LDH so does not appear to be a consequence of continued selection pressure. An alternative explanation may be the decreased sensitivity of the Pf-LDH RDT compared with the HRP2-only RDT for parasite densities ≤1000 parasites/μL, and the shorter half-life of Pf-LDH, or the trend for HRP2-negative parasites to circulate as low density (asymptomatic) infections, potentially increasing the multiplicity of infection.

The purpose of this study was to compare outcomes of different decisions regarding changing RDTs used for diagnosis of symptomatic individuals suffering from malaria. In this context, the actual values of key outcomes are less important than the trends and patterns observed between simulation scenarios. The use of a small population size in the model means the predicted times to key events are likely accelerated compared to regional or country-wide scenarios. We have also taken a singular approach where all antimalaria treatment is preceded by a positive RDT result, an approach which heightens the selection pressure on HRP2-negatiave parasites. This is unlike most settings where providers may be noncompliant with RDT results or patients may purchase antimalarials in the retail sector regardless of RDT results.

Currently it is not possible to validate the patterns observed from the results of this simulation model as there have been no published studies reporting the impact of an RDT change due to the presence of *pfhrp2*-deleted parasites. Eritrea officially changed RDT due to the presence of HRP2-negative parasites in 2017 so provides the potential in the future to measure the impact of changing RDTs. However, Eritrea has highly seasonal malaria which may confound comparison with these model outputs where transmission is assumed to be constant. It will also likely be difficult to validate the observation that persistent HRP2-negative parasites tend to occur in low density infections following the RDT change due to methodological limitations with identifying gene deletions in low density samples. A further possible complication for validating model results against field data in the future is the recent report that many HRP2-detecting RDTs, but not all, can detect *pfhrp2*-negative/*pfhrp3-*positive parasites at 200 parasites/μL [36] meaning the prevalence of false-negative RDTs is likely related to the specific RDT used at a location, combined with the deletion status of both *pfhrp2* and *pfhrp3*.

As with all modelling studies the results are sensitive to the model assumptions. One of the new and key assumptions relates to the recombination of *pfhrp2* when male and female gametes have different genotypes. It is assumed that progeny have equal chances of inheriting each genotype based on the unbiased inheritance patterns seen for *pfhrp2* in the Dd2 and HB3 genetic cross [37]. Any bias in the inheritance of the HRP2-negative phenotype will influence the results of this study, particularly the parasite extinction rate and long-term prevalence of the HRP2-negative parasites. It has also been assumed that deletion of *pfhrp2* does not result in a loss (or gain) of fitness, something which has not been tested in field isolates. The results of this study are also sensitive to the complex interactions between parasite genetics and host immunity, both of which combine to influence parasite transmission and survival.

The model does not consider the role that clinical presentation for non-malarial fevers could play in the facilitating or hindering the spread of HRP2-negative parasites. It is not obvious whether inclusion of testing by RDT of non-malarial fever, and subsequent treatment of positive cases, would significantly impact the results presented here. There is likely little impact when the probability of a host presenting clinically with a non-malarial fever being co-infected with malaria is low, irrespective of the HRP2 phenotype of the infecting parasite. In settings where the probability of co-infection is higher, there may be an impact on the timing of events depending on the prevalence of non-malarial fevers tested by RDT, the prevalence of HRP2-negative parasites and the multiplicity of infection in hosts, but it is unlikely that the overall patterns observed following a change in RDT, and the relative comparisons between scenarios would be impacted.

This study has demonstrated that switching the diagnostic RDT away from a HRP2-only RDT when the prevalence of HRP2-negative parasites reaches pre-defined thresholds restores the ability to correctly diagnose symptomatic malaria infections in a simulated population. However, it does not automatically lead to the extinction of HRP2-negative parasites, or return to prevalence and transmission levels equivalent to those prior to the introduction of the HRP2-negative parasite. While this is not ideal, failure to move away from HRP2-only RDTs leads to near fixation of these parasites in the population and the inability to correctly diagnose symptomatic cases. Overall, these results suggest that once established *pfhrp2-*deleted parasites are likely to become a significant component of *P*. *falciparum* parasite populations, and that long-term strategies are needed for diagnosis and surveillance that do not rely solely on HRP2.

## Supporting information

S1 Table Proportion of simulations for Scenarios 1 to 4 where the initial HRP2*-*negative parasite becomes established in population, eventually resulting in a change away from the HRP2-only RDT in Scenarios 1, 2 and 3. (DOCX)

S2 Table Average time (days) between introduction of HRP2-negative parasite and RDT switch in Scenarios 1 to 3 in low, moderate-low and moderate endemic simulations. Combined results from simulations switching to a Pf-LDH RDT and a HRP2/Pf-LDH RDT are presented.
(DOCX)

S1 Fig Kaplan-Meier survival curves of HRP2-negative parasite survival within the community following the RDT switch in low endemic (top), moderate-low endemic (middle) and moderate endemic (bottom) settings. Green line: Scenario 1; blue line: Scenario 2; red line: Scenario 3.
(TIF)

S2 Fig Simulation trajectories for a period of 20 years (7,300 days) for the proportion of all asexual infections caused by HRP2-negative parasites in a village where 50% of symptomatic patients seek diagnosis and treatment for falciparum malaria and the RDT is changed to a Pf-LDH RDT at a 50% false-negative threshold. Vertical dashed line represents RDT change day.
(TIF)

S3 Fig Simulation trajectories for a period of 20 years (7,300 days) for the proportion of all asexual infections caused by HRP2-negative parasites in a village where 50% of symptomatic patients seek diagnosis and treatment for falciparum malaria and the RDT is changed to a HRP2/Pf-LDH RDT at a 25% (Scenario 1b, top), 50% (Scenario 2b, middle) or 75% (Scenario 3b, bottom) false-negative threshold. Vertical dashed line represents RDT change day.
(TIF)

S4 Fig Simulation trajectories for a period of 20 years (7,300 days) for the proportion of all asexual infections caused by HRP2-negative parasites in a village with moderate-low transmission where 80% of symptomatic patients seek diagnosis and treatment for falciparum malaria and a Pf-LDH RDT is used after the RDT change. Scenarios 5 (left) and 6 (right) have a 25% and 75% false-negative change threshold, respectively. Vertical dashed line represents RDT change day.
(TIF)

S5 Fig 20-year simulation trajectories for prevalence of asexual infections (>100 parasites/μL) in a village of 400 people where 50% of symptomatic patients seek diagnosis and treatment for falciparum malaria and a Pf-LDH RDT is used after the RDT change at 50% false-negative threshold. Prevalence represents a census of the population every 50 days. Vertical dashed line: RDT change day; light blue horizontal: 75^th^ percentile of prevalence in 500 days before introduction of HRP2-negative parasite.
(TIF)

S6 Fig Simulation trajectories for prevalence of asexual infections (>100 parasites/μL) (top) and EIR (bottom) over a period of 20 years in a village with moderate-low transmission where 80% of symptomatic patients seek diagnosis and treatment and Pf-pLDH RDT is used after the RDT change. Vertical dashed line: RDT change day; horizontal line: 75^th^ percentile of prevalence (top) or EIR (bottom) in the 500 days before introduction of HRP2-negative parasite.
(TIF)
