# Supplementary material for: Modelling the epidemiology of malaria and spread of HRP2-negative Plasmodium falciparum following the replacement of HRP2-detecting rapid diagnostic tests
Source: PLOS Glob Public Health. 2022 Jan 4;2(1):e0000106. doi: 10.1371/journal.pgph.0000106 (PMC10021339; doi:10.1371/journal.pgph.0000106)
Supplement: S1 Table — (DOCX) [file pgph.0000106.s001.docx]

S1 Table. Proportion of simulations for Scenarios 1 to 4 where the initial HRP2*-*negative parasite becomes established in population, eventually resulting in a change away from the HRP2-only RDT in Scenarios 1, 2 and 3

|  | Proportion of simulations where HRP2-negative parasites become established (n=200 for each scenario and transmission level) | | | | | | | p-value^a^ |
| --- | --- | --- | --- | --- | --- | --- | --- | --- |
|  | Scenario 1a | Scenario 1b | Scenario 2a | Scenario 2b | Scenario 3a | Scenario 3b | Scenario 4 |  |
| Low endemic | 0.805 | 0.810 | 0.845 | 0.820 | 0.805 | 0.865 | 0.825 | 0.646 |
| Moderate-low endemic | 0.785 | 0.755 | 0.800 | 0.775 | 0.735 | 0.755 | 0.780 | 0.771 |
| Moderate endemic | 0.625 | 0.620 | 0.555 | 0.620 | 0.625 | 0.635 | 0.675 | 0.383 |

^a^ p-value from Pearson Chi-square test comparing proportion of simulations where HRP2*-*negative parasite became established between scenarios
