# Supplementary material for: Modelling the epidemiology of malaria and spread of HRP2-negative Plasmodium falciparum following the replacement of HRP2-detecting rapid diagnostic tests
Source: PLOS Glob Public Health. 2022 Jan 4;2(1):e0000106. doi: 10.1371/journal.pgph.0000106 (PMC10021339; doi:10.1371/journal.pgph.0000106)
Supplement: S2 Table — Combined results from simulations switching to a Pf-LDH RDT and a HRP2/Pf-LDH RDT are presented. (DOCX) [file pgph.0000106.s002.docx]

S2 Table. Average time (days) between introduction of HRP2-negative parasite and RDT switch in Scenarios 1 to 3 in low, moderate-low and moderate endemic simulations. Combined results from simulations switching to a Pf-LDH RDT and a HRP2/Pf-LDH RDT are presented.

|  | | Scenario 1 (25% threshold) | Scenario 2 (50% threshold) | Scenario 3 (75% threshold) |
| --- | --- | --- | --- | --- |
| Low endemic | Mean  (95% CI) | 351.3  (340.7 – 362.0) | 380.0  (369.9 - 390.2) | 460.2  (448.6 - 471.8) |
|  | Median  (Q1 – Q3) | 332.0  (292.0 – 394.0) | 362.0  (314.0 - 429.5) | 447.5  (386.8 – 518.0) |
|  | n* | 323 | 333 | 334 |
| Moderate-low endemic | Mean  (95% CI) | 346.1  (335.9 - 356.3) | 430.9  (419.9 - 441.9) | 551.7  (538.6 - 564.7) |
|  | Median  (Q1 – Q3) | 326.5  (286.0 – 390.0) | 409.0  (362.0 – 479.0) | 541.0  (468.8 - 609.3) |
|  | n* | 308 | 315 | 298 |
| Moderate endemic | Mean  (95% CI) | 433.8  (421.9 - 445.8) | 573.3  (560.5 – 586.0) | 1591.4  (1512.9 - 1669.9) |
|  | Median  (Q1 – Q3) | 412.0  (370.0 – 490.0) | 559.0  (507.0 – 629.0) | 1609.5  (1121.8 – 1995.0) |
|  | n* | 249 | 235 | 252 |

* represents the number of simulations in which the HRP2-negative parasite became established in the population, resulting in an RDT switch
