# Supplementary figures and images for: Modelling the epidemiology of malaria and spread of HRP2-negative Plasmodium falciparum following the replacement of HRP2-detecting rapid diagnostic tests
Source: PLOS Glob Public Health. 2022 Jan 4;2(1):e0000106. doi: 10.1371/journal.pgph.0000106 (PMC10021339; doi:10.1371/journal.pgph.0000106)

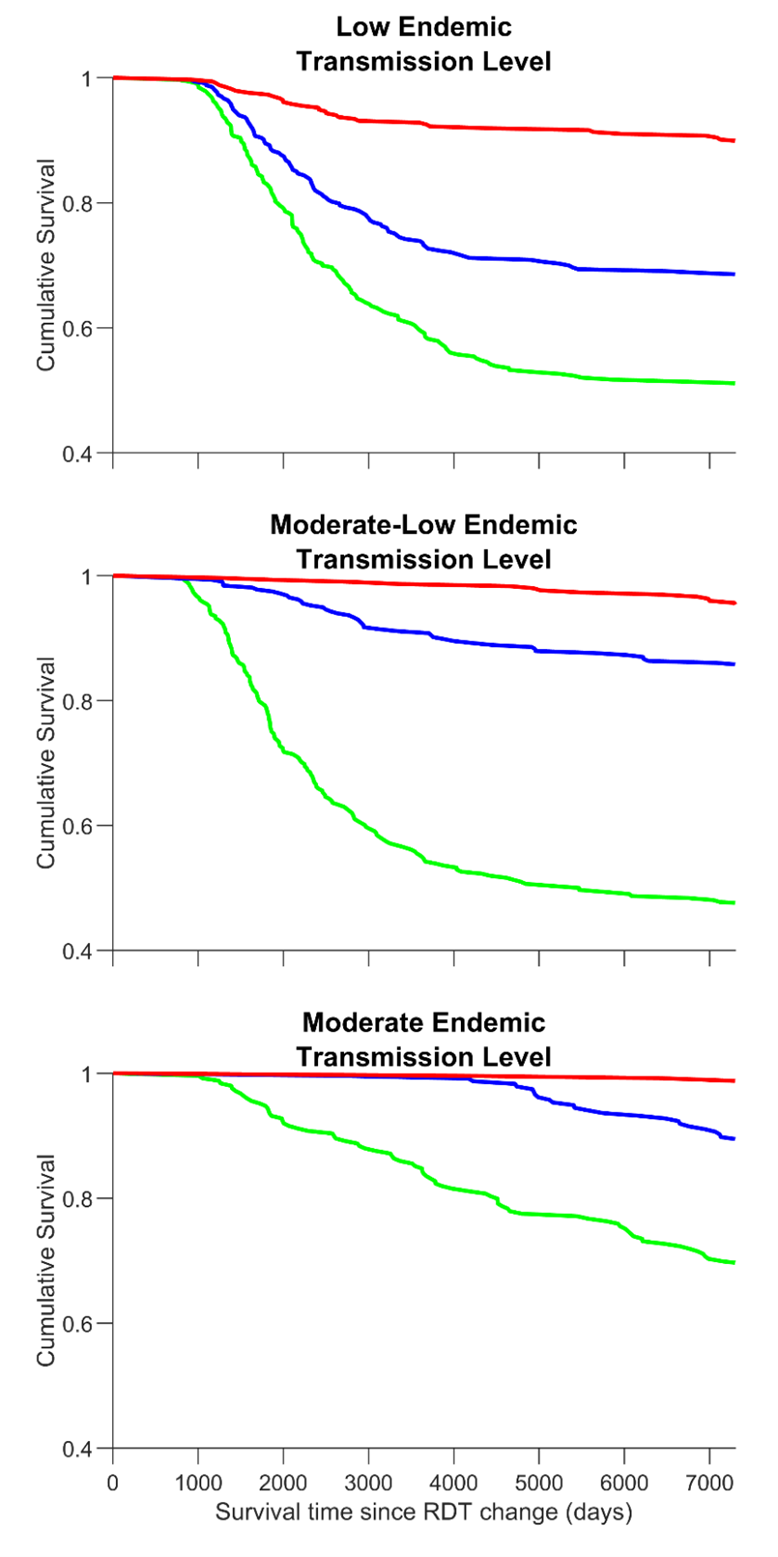

Supplement: S1 Fig — Green line: Scenario 1; blue line: Scenario 2; red line: Scenario 3. (TIF) [file pgph.0000106.s003.tif]

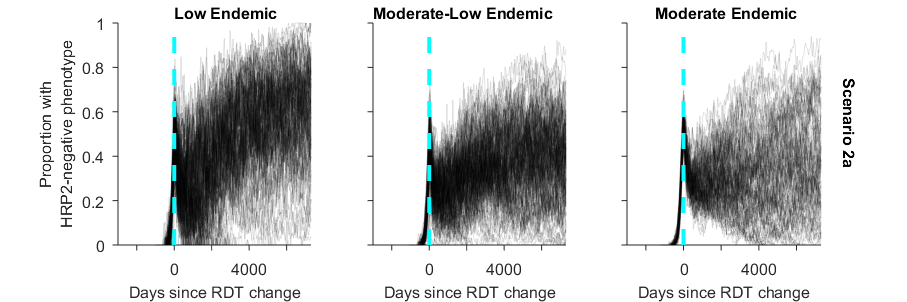

Supplement: S2 Fig — Vertical dashed line represents RDT change day. (TIF) [file pgph.0000106.s004.tif]

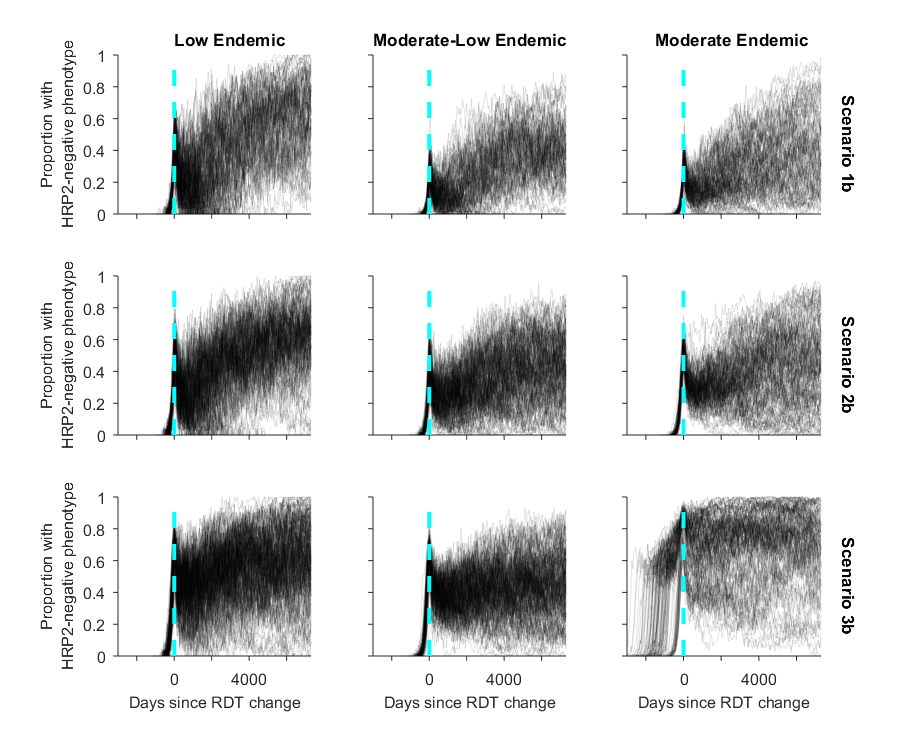

Supplement: S3 Fig — Vertical dashed line represents RDT change day. (TIF) [file pgph.0000106.s005.tif]

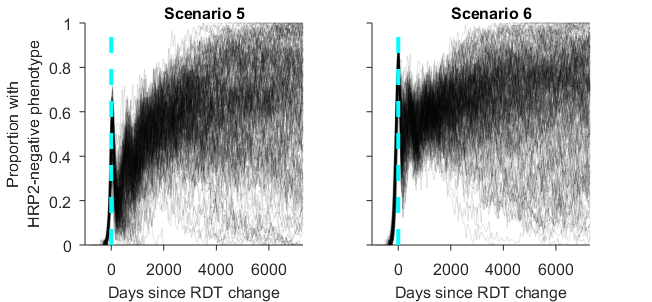

Supplement: S4 Fig — Scenarios 5 (left) and 6 (right) have a 25% and 75% false-negative change threshold, respectively. Vertical dashed line represents RDT change day. (TIF) [file pgph.0000106.s006.tif]

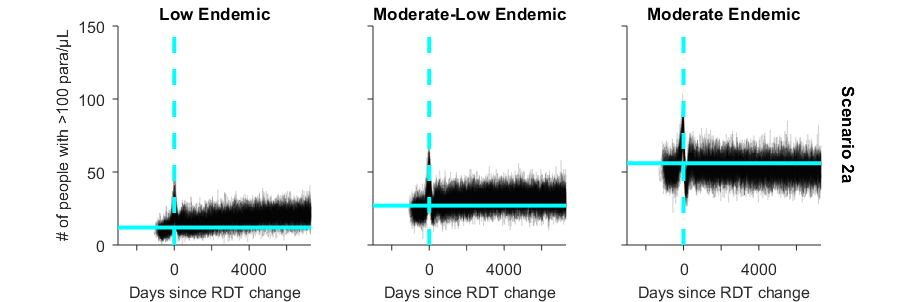

Supplement: S5 Fig — Prevalence represents a census of the population every 50 days. Vertical dashed line: RDT change day; light blue horizontal: 75th percentile of prevalence in 500 days before introduction of HRP2-negative parasite. (TIF) [file pgph.0000106.s007.tif]

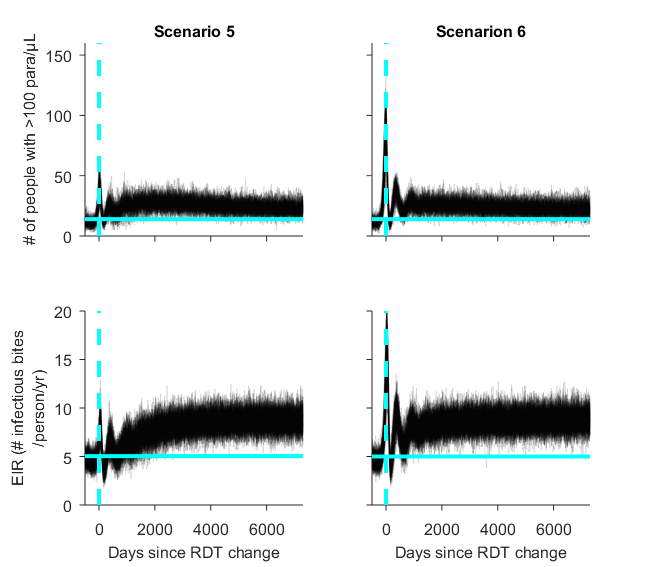

Supplement: S6 Fig — Vertical dashed line: RDT change day; horizontal line: 75th percentile of prevalence (top) or EIR (bottom) in the 500 days before introduction of HRP2-negative parasite. (TIF) [file pgph.0000106.s008.tif]
